# Modeling the current distribution suitability and future dynamics of *Culicoides imicola* under climate change scenarios

**DOI:** 10.7717/peerj.12308

**Published:** 2021-10-29

**Authors:** Hongyan Gao, Long Wang, Jun Ma, Xiang Gao, Jianhua Xiao, Hongbin Wang

**Affiliations:** 1 College of Veterinary Medicine, Northeast Agricultural University, Harbin, People’s Republic of China; 2 Key Laboratory of the Provincial Education Department of Heilongjiang for Common Animal Disease Prevention and Treatment, College of Veterinary Medicine, Northeast Agricultural University, Harbin, People’s Republic of China

**Keywords:** *Culicoides imicola*, Global climate change, African horse sickness, Ecological niche model

## Abstract

**Background:**

African horse sickness, a transboundary and non-contagious arboviral infectious disease of equids, has spread without any warning from sub-Saharan Africa towards the Southeast Asian countries in 2020. It is imperative to predict the global distribution of *Culicoides imicola* (*C. imicola*), which was the main vector of African horse sickness virus.

**Methods:**

The occurrence records of *C. imicola* were mainly obtained from the published literature and the Global Biodiversity Information Facility database. The maximum entropy algorithm was used to model the current distribution suitability and future dynamics of *C. imicola* under climate change scenarios.

**Results:**

The modeling results showed that the currently suitable habitats for *C. imicola* were distributed in most of the southern part areas of America, southwestern Europe, most of Africa, the coastal areas of the Middle East, almost all regions of South Asia, southern China, a few countries in Southeast Asia, and the whole Australia. Our model also revealed the important environmental variables on the distribution of *C. imicola* were temperature seasonality, precipitation of coldest quarter, and mean temperature of wettest quarter. Representative Concentration Pathways (RCPs) is an assumption of possible greenhouse gases emissions in the future. Under future climate change scenarios, the area of habitat suitability increased and decreased with time, and RCP 8.5 in the 2070s gave the worst prediction. Moreover, the habitat suitability of *C. imicola* will likely expand to higher latitudes. The prediction of this study is of strategic significance for vector surveillance and the prevention of vector-borne diseases.

## Introduction

*Culicoides* biting midges play an important role in the spread of vector-borne infectious diseases worldwide, transmitting disease agents to humans, domestic and wild animals. Among them, African horse sickness is a transboundary and non-contagious infectious disease of equids and is one of the most lethal equine virus infections known. The pathogenic virus is African horse sickness virus (AHSV), which belongs to the genus *Orbivirus*, the family *Reoviridae* (Howell, 1962). The World Organization for Animal Health classifies African horse sickness as a listed notifiable disease (OIE, 2021).

The epidemic area and seasonality of African horse sickness occurrence are related to vector epidemiology (Robin, 2019). At present, Meiswinkel & Paweska (2003) have shown that *Culicoides bolitinos* is a proven vector of AHSV in South Africa. *Culicoides imicola* (*C. imicola*) is another important vector for field transmission of AHSV (Mellor & Boorman, 1995). It is widely distributed in most of the inhabited world, including Africa, southern Europe, and southern Asia, which exist *C. imicola* around the years, and are also potential risk areas of African horse sickness occurrence (Guichard et al., 2014; Meiswinkel, 1989).

African horse sickness virus is endemic in sub-Saharan Africa, and it periodically invaded Europe and Asia (Carpenter et al., 2017). In 2020, Thailand and Malaysia successively reported the first incidence of African horse sickness, the first outbreak caused by AHSV-1 outside sub-Saharan Africa (King et al., 2020). The epidemic could pose a major threat to Southeast Asia and even other Asian countries. Currently, the effective way to prevent and control African horse sickness is to control *Culicoides* population.

Under the increasingly severe situation of prevention and control of *Culicoides*-borne diseases in the world, it is essential to better understand the possible geographical dynamics of *Culicoides* vector. Species distribution models can assist in the targeted monitoring and the implementation of controlling programs for disease vector (Liu et al., 2019). Therefore, we used the ecological niche model to evaluate the current global distribution of *C. imicola*, based on occurrence records and bioclimate variables. In addition, our modeling projected the future habitats in the 21st century based on global climate change.

## Materials & methods

### *Culicoides imicola* data and processing

**Figure 1:**
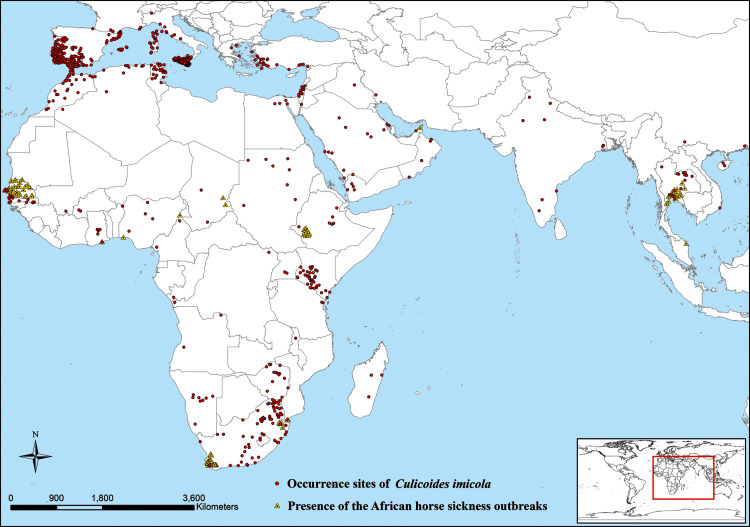
Presence records of *Culicoides imicola* and African horse sickness outbreaks in the world. Presence sites of *Culicoides imicola* (red round points) were taken from the literature and Global Biodiversity Information Facility database. Locations of African horse sickness outbreaks (yellow triangle points) were downloaded from Food and Agriculture Organization of the United Nations.

We obtained the *C. imicola* presence points (*n* = 1,046) from the literature (Duan et al., 2021; Leta et al., 2019; Ye et al., 2019) and the Global Biodiversity Information Facility database (https://www.gbif.org/). All the occurrence data were spatially filtered at 10 km^2^ grid cells (Radosavljevic & Anderson, 2014) to minimize the spatial autocorrelation using the Species Distribution Model Toolbox (Brown, Bennett & French, 2017). Thus, 729 spatially rarefied occurrence records of *C. imicola* were included in the current and future model in this study (Fig. 1).

### Environmental variable collection and processing

To determine the influence of the environmental variables on the *C. imicola* distribution, we considered 19 bioclimate variables as risk factors in the model (Table 1). The current and future climate data were downloaded from the WorldClim website. Representative Concentration Pathways (RCPs) is an assumption of possible greenhouse gases emissions in the future (van Vuuren et al., 2011), which originates from the Intergovernmental Panel on Climate Change Assessment Report. In this study, we chose RCP 2.6, RCP 4.5, and RCP 8.5 to represent the three scenarios of minimum, medium and maximum emissions, respectively. RCPs under the global circulation model of the Beijing Climate Center Climate System version 1.1 were used to explore *C. imicola* distribution in the periods of 2030s (average for 2021–2040), 2050s (average for 2041–2060), and 2070s (average for 2061–2080).

**Table 1:** Description of the variables used in the model.

Variables	Description	Included	Source
bio_1	Annual mean temperature		WorldClim (http://worldclim.org)
bio_2	Mean diurnal range (Mean of monthly (max temp –min temp))	Yes	
bio_3	Isothermality (Bio 2/Bio 7) (×100)		
bio_4	Temperature seasonality (standard deviation × 100)	Yes	
bio_5	Max temperature of warmest month		
bio_6	Min temperature of coldest month		
bio_7	Temperature annual range (Bio 5–Bio 6)		
bio_8	Mean temperature of wettest quarter	Yes	
bio_9	Mean temperature of driest quarter	Yes	
bio_10	Mean temperature of warmest quarter		
bio_11	Mean temperature of coldest quarter		
bio_12	Annual precipitation		
bio_13	Precipitation of wettest month	Yes	
bio_14	Precipitation of driest month	Yes	
bio_15	Precipitation seasonality (Coefficient of Variation)	Yes	
bio_16	Precipitation of wettest quarter		
bio_17	Precipitation of driest quarter		
bio_18	Precipitation of warmest quarter	Yes	
bio_19	Precipitation of coldest quarter	Yes	

**Note:**

The current and future bioclimatic data were obtained from the WorldClim website. After removing multi-collinearity, nine variables were included in the final model.

Variance inflation factors were calculated to avoid the multi-collinearity of environmental variables, it is considered that variables with variance inflation factors greater than 10 were highly collinear (Ma et al., 2020), and each pair of variables should also maintain a correlation value |r| < 0.7. As a result, a total of nine environment variables were included in the model (see Table 1). In ArcGIS 10.2 (Environmental Systems Research Institute, Redlands, CA, USA), all the environmental variables were resampled to the American Standard Code for Information Interchange at a resolution of 2.5 arcminutes.

### MaxEnt model construction

The maximum entropy (MaxEnt) model was used for modelling (http://biodiversityinformatics.amnh.org/open_source/maxent/). MaxEnt model is a machine learning method, which is used to analyze with the presence-only point data (Phillips, Anderson & Schapire, 2006). In the modeling, 25% of the occurrence points were randomly set as test points, and the remaining 75% were training points (Yang et al., 2018). To account for the sampling bias, we created a bias file and 10,000 background points were taken into the MaxEnt models as “pseudo-absence” data (Gao & Ma, 2021; Kramer-Schadt et al., 2013).

The AUC (the area under the receiver operating characteristic curve) assesses the predictive performance of the model. The range of AUC value is 0–1, and a higher value corresponds to a better predictive model (Phillips, Anderson & Schapire, 2006). To evaluate the importance of the environmental variables in modeling, the percent contribution of variables was used as indicators of MaxEnt model.

The result maps were visualized using ArcGIS 10.2 (ESRI Inc., Redlands, CA, USA). According to the Intergovernmental Panel on Climate Change Assessment Report (Manning, 2006), the continuous probability of *C. imicola* distribution was reclassified: unsuitable (less than 0.05), low suitability (0.05–0.33), medium suitability (0.33−0.67), and high suitability (greater than 0.67).

## Results

The AUC value is 0.902 (±0.011) in the current model for *C. imicola* distribution (Fig. S1), indicating the excellent predictive power of the model. The contributions of the environmental variables were shown in Table S1, and the top three variables with greater contribution were important variables. Temperature seasonality was identified as the most important variable for model construction (30.3% contribution), followed by precipitation of coldest quarter (29.5% contribution) and mean temperature of wettest quarter (16.5% contribution).

**Figure 2:**
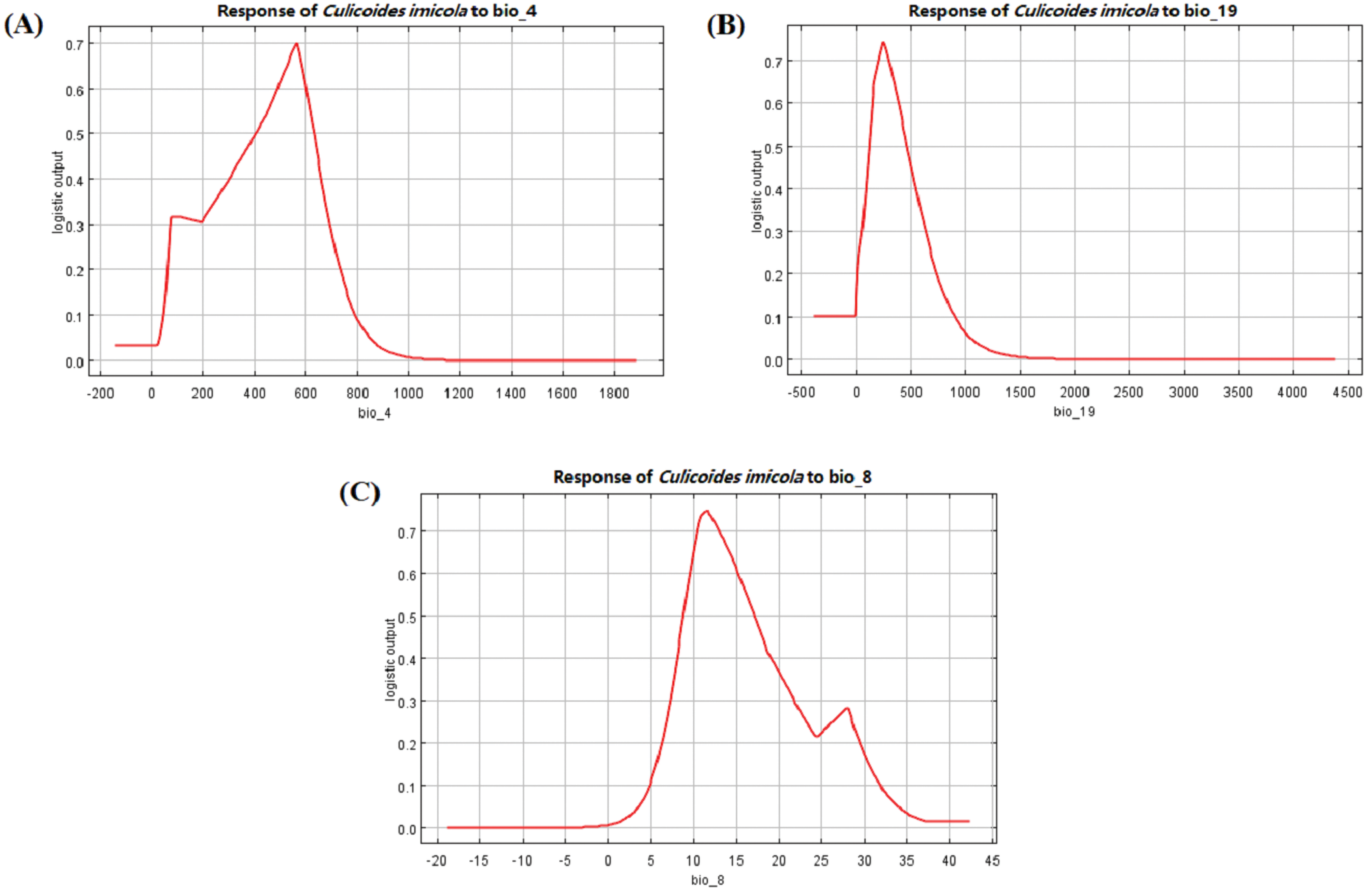
Response curves of variables with greater contribution. The *y*-axis represents the habitat suitability of *Culicoides imicola*, while the *x*-axis of the variables represents the variation range. (A) Bio_4-Temperature seasonality (Unit: °C). (B) Bio_19-Precipitation of coldest quarter (Unit: mm). (C) Bio_8-Mean temperature of wettest quarter (Unit: °C).

The response curves of the variables reflect the environmental requirements of *C. imicola* (Fig. 2). In this non-identifiability of prevalence, when the threshold of habitat suitability (*i.e*., logistic output) was greater than 0.5, the variation range represented by the horizontal axis was the optimal range (Elith et al., 2011). In the response curve of bio_4, temperature seasonality represented the standard deviation of the monthly mean temperature multiplied by 100. When temperature seasonality was about 564 (*i.e*., the standard deviation of the monthly mean temperature is 5.64), the predicted habitat suitability for *C. imicola* was better for values of optimal range from 403 to 631 (Fig. 2A). In the response curve of bio_19, the optimal range for precipitation of coldest quarter was from 115 to 462 mm. When precipitation exceeded 244 mm, the habitat suitability decreased rapidly, and the probability of *Culicoides* distribution was 0 when precipitation exceeded 1,500 mm (Fig. 2B). For bio_8, the optimal range for mean temperature of wettest quarter was from 8.8 to 17 °C. In addition, the habitat suitability of *C. imicola* was highest when the temperature was about 11.5 °C (Fig. 2C).

**Figure 3:**
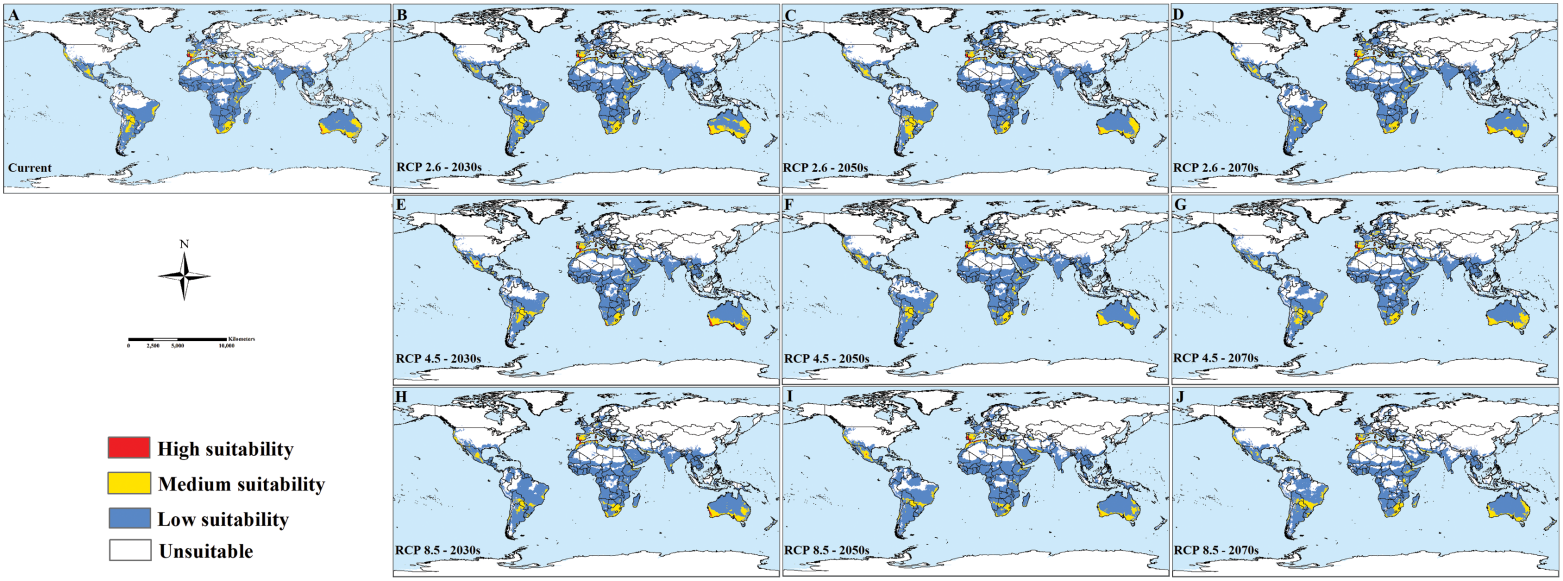
Modelled habitat suitability of *Culicoides imicola* under current and future climate change scenarios. (A) Projection areas in current climate scenarios, (B–J) projection areas in future period. Each color represents the different suitability area of *Culicoides imicola*.

The potential habitat suitability of *C. imicola* under current and future climate scenarios were shown in Fig. 3. In the current climate scenario, southern North America, southern South America, southwestern Europe, most of Africa, the coastal areas of the Middle East, almost all regions of South Asia, southern China, a few countries in Southeast Asia, and the whole Australia were predicted as suitable habitats for *C. imicola* by the MaxEnt model. Among these areas, small parts of southern coastal Europe and northern coastal Africa were high suitability for *C. imicola* distribution. In the future climate scenarios, the predicted areas under different scenarios were generally similar, except for Australia under the RCP 4.5 scenario in the 2030s. However, suitability was expected to increase at high latitudes in the northern hemisphere, such as Norway, Sweden, Finland, and the Kola Peninsula. Moreover, in the low and middle latitudes of the southern hemisphere, the medium suitability areas in South America and high suitability areas in the western and southern coasts of Australia were predicted to decrease significantly.

**Figure 4:**
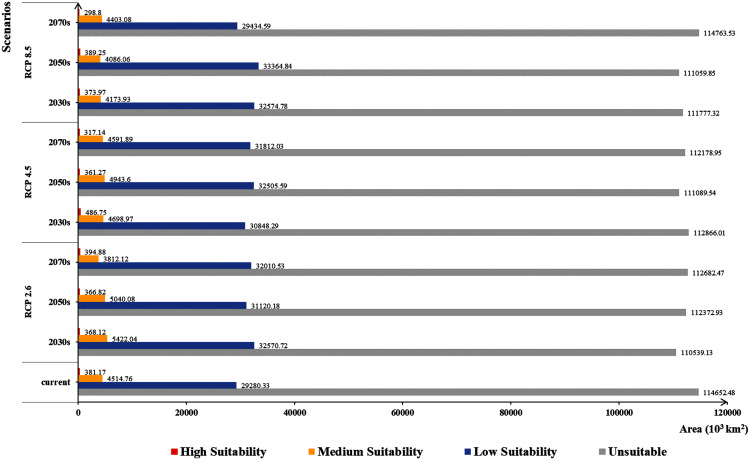
Bar graph of habitat suitability area under different climate change scenarios. These bars are the area from current and future climate scenario maps (Unit: 10^3^ km^2^). Each color represents the different suitability area of *Culicoides imicola*.

The areas of different habitat suitability under current and future climate scenarios were shown in Fig. 4, and the current suitable area of *C. imicola* was estimated to be about 34,176,260 km^2^. Except for the 2070s under RCP 8.5, the unsuitable areas for *C. imicola* will decrease continuously under all future climate scenarios. However, compared with the current climate scenario, the areas of low suitability will continue to increase in the future. The change of medium suitability areas under the RCP 2.6 scenario was higher, while the change of high suitability areas under the RCP 4.5 scenario in the 2030s and RCP 8.5 scenario in 2070s were higher (the increments were 27.70% and 21.61%, respectively). It is worth noting that the suitable areas under the RCP 8.5 scenario hardly increased in the 2070s, but decreased sharply.

## Discussion

Temperature and rainfall are determining factors in the activity, abundance, and survival of *Culicoides* (Mellor, Boorman & Baylis, 2000). Previous studies have demonstrated that temperature is positively correlated with the *Culicoides* activity, and adult vector activity was suppressed at low temperatures (Murray, 1987; Searle et al., 2014). Adult *Culicoides* activity was related to seasonality (Carpenter et al., 2011; Sanders et al., 2019). At the same time, high temperature favored larval development leading to faster population growth (Kitaoka, 1982; Vaughan & Turner, 1987). Furthermore, at high temperature, the vector virogenesis rates were high (Verhoef, Venter & Weldon, 2014; Wittmann, Mello & Baylis, 2002). However, laboratory studies have shown that individual *Culicoides* survived for a relatively short lifespan at very high temperature (Wellby, Matthew & Peter, 1996; Wittmann, Mello & Baylis, 2002).

On the other hand, rainfall can also influence the activity of *C. imicola*, which will decrease at low moisture levels (Walker, 1977). In Australia, after rainfall, there was an increase in the feed time of many *Culicoides* midges. The feeding frequency influences the host-biting rates; therefore, the population transformed to one capable of explosive transmission (Murray, 1986). At a suitable temperature, the abundance of the vector is often more closely related to rainfall. Diarra found that the largest amount of *C. imicola* abundance appeared in the year of the greatest rainfall in Senegal (Diarra et al., 2014). In addition, rainfall influences the spread of African horse sickness by governing the availability of larval habitat and regulating the survival and dispersal of adult *Culicoides* (Purse et al., 2015). The water content can determine the suitable semiaquatic habitat for the larva (Meiswinkel, 1997), and more rain may produce more suitable habitats. However, if the habitats are flooded, *C. imicola* will drown (Nevill, 1967).

The predicted map showed that the neighboring countries, India, Myanmar, Southern China, Laos, Cambodia, and Vietnam were all in the suitable areas for *C. imicola*. Identification of such regions is related to biosecurity purposes. Bluetongue virus (BTV) belongs to the same genus as AHSV and mainly infects ruminants. Recently, cases of BTV infection have been confirmed in *C. imicola* in Yunnan Province of China (Duan et al., 2021), suggesting that introducing AHSV infection types into disease-free areas is possible, therefore suitable areas of the vector should be strictly monitored.

In the future scenario, the habitat suitability of *C. imicola* will likely expand to higher latitudes, and the predicted map even showed that there were low suitability areas in high latitudes in Norway, Sweden, Finland, and the Kola Peninsula. Rawlings et al. (1997) also proposed that global climate change may cause *C. imicola* to expand northward. Moreover, it is worth noting that the Americas and Australasia have large areas of low suitability and medium suitability, and if *C. imicola* were translocated to these areas, there are increasing risks for *C. imicola* to expand the activity range. This will lead to a wider geographical distribution of the AHSV, thereby increasing the risk of exposure to diseases.

The surveillance of *C. imicola* is of great significance for the prevention and control of African horse sickness because AHSV was transmitted to domestic and wild populations through *C. imicola* biting. In domestic populations, the horses of AHSV infection have obvious clinical signs, a short period of viremia, and high mortality (Robin, 2019; Wilson et al., 2009). In wild populations, zebra played a crucial role in the persistence of AHSV in Africa and was considered the natural virus reservoir (Mellor & Hamblin, 2004). Zebra rarely showed clinical signs, but the period of viremia can extend approximately 40 days (Barnard et al., 1994). In 2020, African horse sickness was reported in Southeast Asia, and vaccination was the most effective way to control African horse sickness. Before the vaccine development, the priority measures were to prevent vector-host interaction. Stabling of horses overnight can protect horses from African horse sickness because *C. imicola* and *Culicoides bolitinos* were less reluctant to enter enclosed space (Wilson et al., 2009). Stabling was also a practical way as part of quarantine measures related to the intercontinental transport of horses (Page et al., 2015). Therefore, multiple integrated measures (such as staling of horses, clearance of larval habitat, insecticides, etc.) may have a greater impact on AHSV transmission (Carpenter et al., 2017).

This study still has a limitation, for most of *C. imicola* occurrence data were obtained from the published literature, which might suffer from underreporting. However, the results could be related to differences in reporting rather than the true ecological habits of *Culicoides*. In the future, it will be interesting to model the impact of the geographical expansion of *Culicoides* on disease distribution. Simultaneously, it is of great significance if the prediction model includes *Culicoides* survivability under high temperatures.

## Conclusions

Based on *C. imicola* occurrence records and bioclimatic variables, the current and future suitable habitat of *C. imicola* all over the world was modeled using MaxEnt model. Three bioclimatic variables were revealed to have important effects on *C. imicola* distribution. In the 21st century, the habitat suitability of *C. imicola* may be different with climate change. The prediction of this study is of strategic significance for vector surveillance and the prevention of vector-borne diseases.

## Supplemental Information

10.7717/peerj.12308/supp-1 Supplemental Information 1 Supplemental Figure and Table.

## Additional Information and Declarations

### Data Availability

The following information was supplied regarding data availability: The Occurrence data of *Culicoides imicola* is available at Global Biodiversity Information Facility database (https://www.gbif.org/occurrence/search?taxon_key=1632059), and the bioclimatic data is available at WorldClim website (current data : https://worldclim.org/data/worldclim21.html; future data: https://www.worldclim.org/data/v1.4/cmip5_2.5m.html).
