# Supplementary material for: Modeling the current distribution suitability and future dynamics of Culicoides imicola under climate change scenarios
Source: PeerJ. 2021 Oct 29;9:e12308. doi: 10.7717/peerj.12308 (PMC8559603; doi:10.7717/peerj.12308)
Supplement: Supplemental Information 1 [file peerj-09-12308-s001.docx]

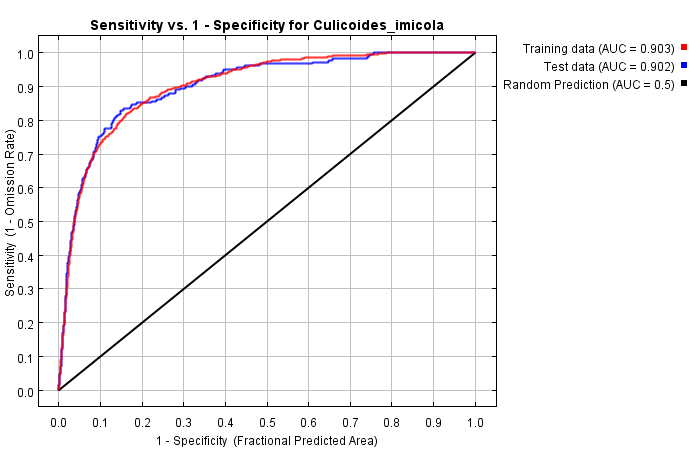


Figure S1 ROC curve of the current *C.imicola* distribution model.

Table S1 Variable contributions in MaxEnt model of current *C.imicola* distribution

| Variable | | Percent contribution (%) | Permutation importance (%) |
| --- | --- | --- | --- |
| bio_4 | Temperature seasonality | 30.3 | 46.4 |
| bio_19 | Precipitation of coldest quarter | 29.5 | 10 |
| bio_8 | Mean temperature of wettest quarter | 16.5 | 7.4 |
| bio_14 | Precipitation of driest month | 9.5 | 6.6 |
| bio_9 | Mean temperature of driest quarter | 6 | 15.8 |
| bio_13 | Precipitation of wettest month | 5.7 | 8 |
| bio_18 | Precipitation of warmest quarter | 1.4 | 1 |
| bio_15 | Precipitation seasonality | 0.7 | 2.7 |
| bio_2 | Mean diurnal range | 0.5 | 2.2 |
